# Supplementary material for: In vitro reconstitution defines the mechanistic basis of HSET motor activity regulation by IntraFlagellar Transport proteins
Source: Commun Biol. 2026 Apr 10;9:808. doi: 10.1038/s42003-026-10007-3 (PMC13265731; doi:10.1038/s42003-026-10007-3)
Supplement: Supplementary file 2 — Supplementary Informations [file 42003_2026_10007_MOESM2_ESM.pdf]

SUPPLEMENTARY FIGURES

In vitro reconstitution defines the mechanistic basis of HSET motor activity regulation by IntraFlagellar Transport proteins

Audrey Guesdon, Valérie Simon, Ron Siaden-Ortega, Juliette Van Dijk, Julien Marcoux, Bénédicte Delaval and Benjamin Vitre

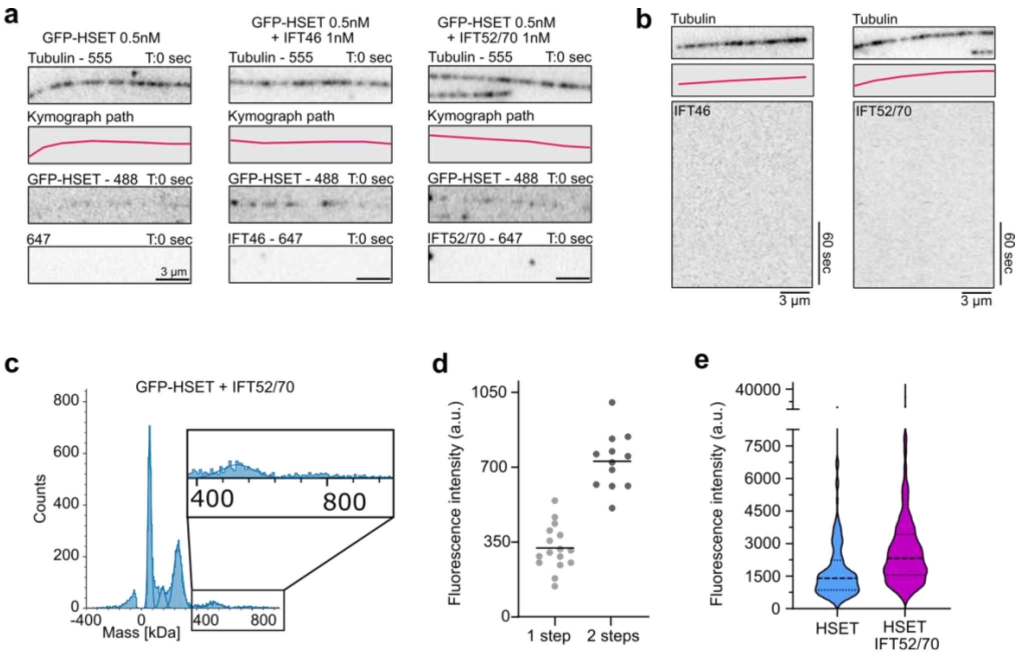

Supplementary Figure 1.

a. Images of the field of view from Movies 1, 2 and 3 used to generate the kymographs in Fig. 1d. **b.** Left: image of a field of view of a microtubule (top) use to generate a kymograph of IFT46 alone (bottom). IFT46 alone does not bind to microtubules. Right: image of a field of view of a microtubule (top) use to generate a kymograph of IFT52/70 alone (bottom). The IFT52/70 dimer alone does not bind to microtubules. **c.** Mass photometry analysis of GFP-HSET dimer (2.5 nM) incubated with IFT52/70 dimer (5 nM) as presented in Figure 2e with a zoom-in inset of the event between 400 and 960 kDa showing multiple counts between 720 and 800 kDa. **d.** Dot plot of the fluorescence signal of GFP-HSET particles bleaching in one or two steps allowing to identify the average fluorescence intensity of a GFP-HSET dimer (two GFP, two steps). Bars indicate average signal. **e**. Violin plot of GFP-HSET individual particles fluorescent signal. Bars indicate median and quartiles. N=127 for HSET condition and 591 for HSET-IFT52/70 condition.

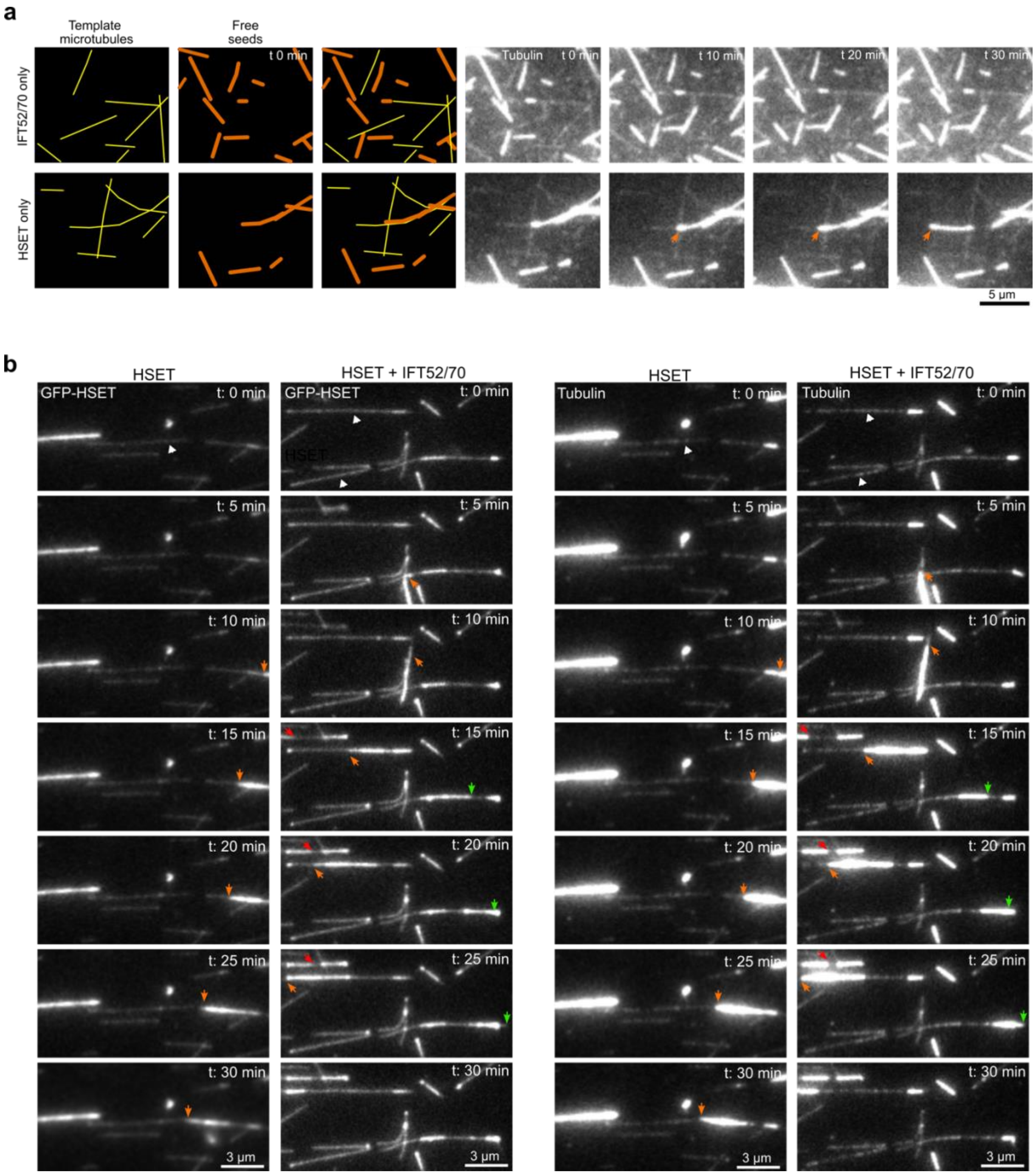

Supplementary Figure 2.

a. Top, schematic and representative TIRF images of free microtubule seeds and template microtubules in presence of IFT52/70 alone. No events of seeds bundling and sliding on template microtubules were observed. Bottom, schematic and representative TIRF images of free microtubules seeds bundling and sliding along template microtubules in presence of GFP-HSET. Orange arrowhead indicate a sliding seed. **b.** Still images corresponding to movies 4 and 5. The two left columns, show the GFP-HSET channel (movie 4) and the two right columns show the tubulin channel (movie 5). The free microtubule seeds are indicated with orange,

red and green arrowheads. Examples of microtubule templates are indicated with white arrowheads. The conditions are: HSET alone (50 nM) or HSET (50 nM) + IFT52/70 (100 nM).

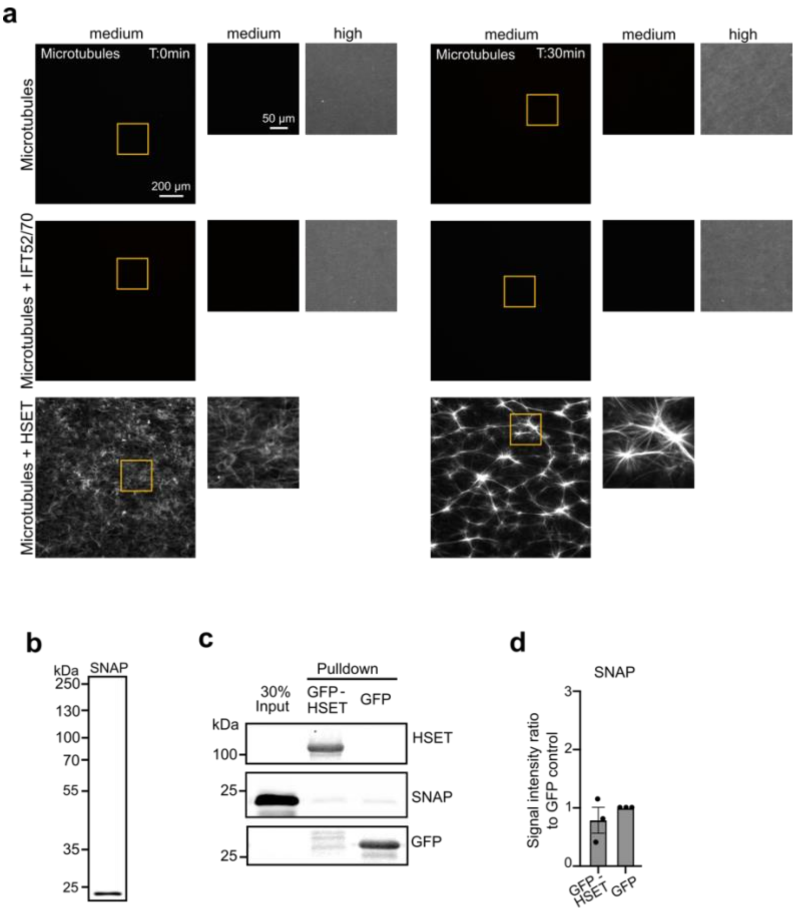

Supplementary Figure 3.

a. Still images from movie showing that IFT52/70 alone cannot form active microtubule networks. The positive control, HSET, displays the same images as the one shown in Fig. 5 a. Insets are the 3x magnification of the orange square in the main field of view. Medium and High refer to contrast and intensity. Even with high contrast and intensity no bundle or active network are observed with IFT52/70 alone. **b.** Coomassie blue staining of purified SNAP-tag. **c.** Western-blot of a GFP-Trap pull down of SNAP-tag. **d**. Quantification of the ratio SNAP-tag pulled-down by GFP-HSET compared to a GFP alone control. Bars represent the average of three independent experiments error bars s.e.m.

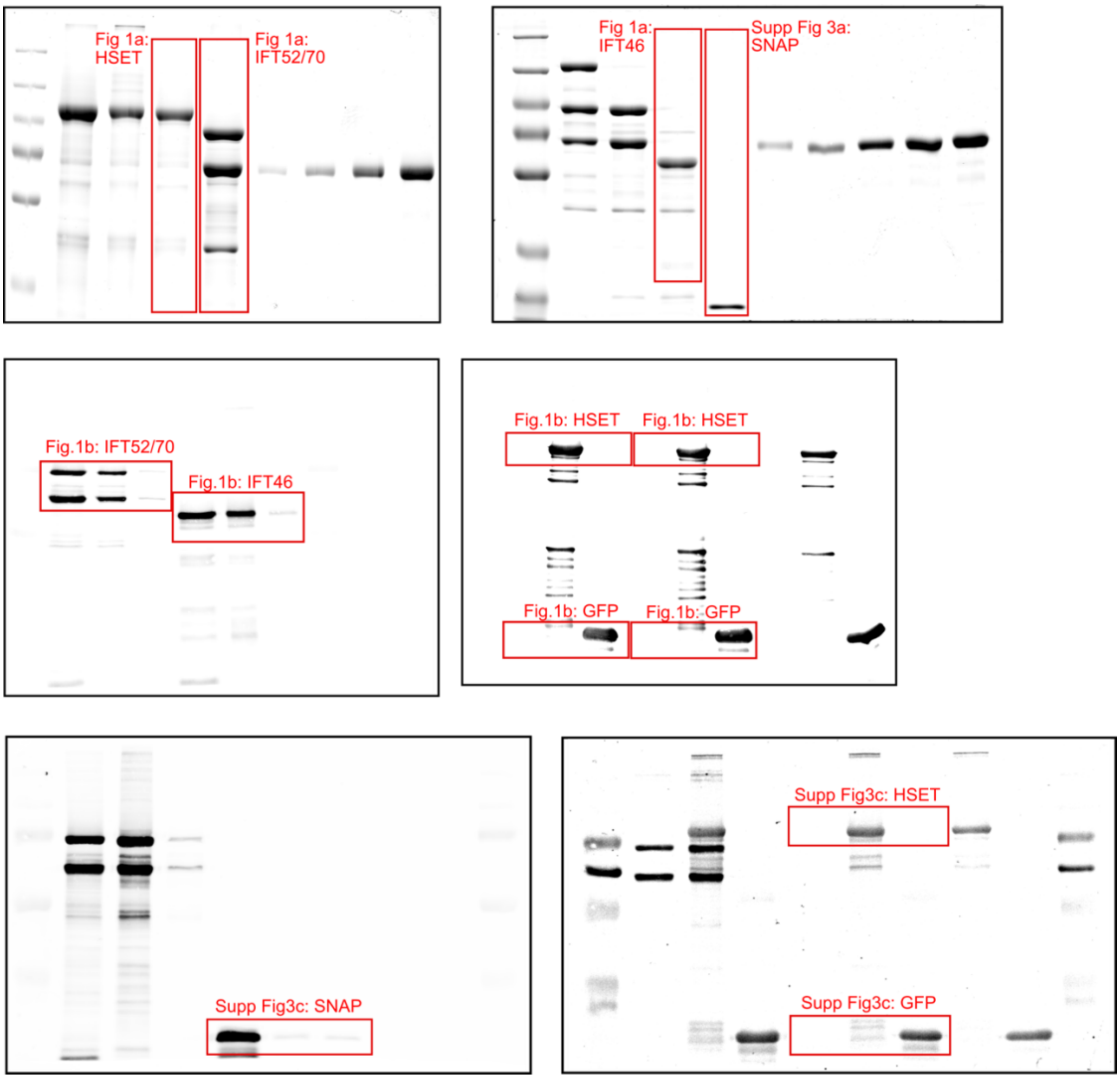

Supplementary Figure 4. Uncropped images of Coomassie staining and western blots presented in Figures 1 and Supplementary Figure 3.
