# Supplementary material for: In vitro reconstitution defines the mechanistic basis of HSET motor activity regulation by IntraFlagellar Transport proteins
Source: Commun Biol. 2026 Apr 10;9:808. doi: 10.1038/s42003-026-10007-3 (PMC13265731; doi:10.1038/s42003-026-10007-3)
Supplement: Supplementary file 3 — Description of Additional Supplementary Files [file 42003_2026_10007_MOESM3_ESM.pdf]

Description of Additional Supplementary File

File name: Supplementary Data 1
Description: The source data underlying the figures are provided in Supplementary Data 1

File name: Supplementary Movie 1
Description: **TIRF microscopy movie of GFP-HSET particle on microtubules, HSET + IFT52/70 condition.**

The field of view corresponds to Supplementary Fig. 1a. Time interval is 1.4 sec. The movie runs at 10 fps. The movie shows the tubulin channel (555 nm), the GFP-HSET channel (488 nm) and the IFT52/70 (647 nm) channel from top to bottom. Scale bar 3 µm.

File name: Supplementary Movie 2
Description: **TIRF microscopy movie of GFP-HSET particle on microtubules, HSET alone condition.**

The field of view corresponds to Supplementary Fig. 1a. Time interval is 1.4 sec. The movie runs at 10 fps. The movie shows the tubulin channel (555 nm), the GFP-HSET channel (488 nm) and the 647 nm channel from top to bottom. Scale bar 3 µm.

File name: Supplementary Movie 3
Description: **TIRF microscopy movie of GFP-HSET particle on microtubules, HSET + IFT46 condition.**

The field of view corresponds to Supplementary Fig. 1a. Time interval is 1.4 sec. The movie runs at 10 fps. The movie shows the tubulin channel (555 nm), the GFP-HSET channel (488 nm) and the IFT46 (647 nm) channel from top to bottom. Scale bar 3 µm.

File name: Supplementary Movie 4
Description: **TIRF microscopy movie of a microtubule seed sliding on a microtubule template in absence or presence of GFP-HSET**

The field of view corresponds to Supplementary Fig. 2b left two columns. Left panel HSET alone condition, right panel HSET +IFT52/70 condition. GFP-HSET (488 nm) is visualized on this movie. Time interval is 1 min. The movie runs at 9 fps

File name: Supplementary Movie 5
Description: **TIRF microscopy movie of a microtubule seed sliding on a microtubule template in absence or presence of GFP-HSET**

The field of view corresponds to Supplementary Fig. 2b right two columns. Left panel HSET alone condition, right panel HSET +IFT52/70 condition. Tubulin (555 nm) is visualized on this movie. Time interval is 1 min. The movie runs at 9 fps.

File name: Supplementary Movie 6
Description: **Wide field epifluorescence microscopy movie of an active microtubule network organizing over time upon the activity of GFP-HSET.**

The field of view corresponds to Fig. 5a top row. Tubulin is visualized on this movie. Time interval is 1.5 min. The movie runs at 10 fps. Scale bar 200 μm.

File name: Supplementary Movie 7
Description: **Wide field epifluorescence microscopy movie of an active microtubule network organizing over time upon the activity of GFP-HSET with IFT52/70.**

The field of view corresponds to Fig. 5a bottom row. Tubulin is visualized on this movie. Time interval is 1.5 min. The movie runs at 10 fps. Scale bar 200 μm.
