# Supplementary material for: In vitro reconstitution defines the mechanistic basis of HSET motor activity regulation by IntraFlagellar Transport proteins
Source: Commun Biol. 2026 Apr 10;9:808. doi: 10.1038/s42003-026-10007-3 (PMC13265731; doi:10.1038/s42003-026-10007-3)
Supplement: Supplementary file 1 — Transparent Peer Review file [file 42003_2026_10007_MOESM1_ESM.pdf]

nature portfolio

Peer Review File

In vitro reconstitution defines the mechanistic basis of HSET motor activity regulation by IntraFlagellar Transport proteins

Corresponding Author: Mr Benjamin Vitre

This file contains all reviewer reports in order by version, followed by all author rebuttals in order by version.

Version 0:

Reviewer comments:

Reviewer #1

(Remarks to the Author)
In the current manuscript the authors examine the mechanisms by which the IFT52/70 complex stimulates HSET activity by enhancing processivity and inducing oligomerization of HSET in vitro. They postulate that this mechanism is critical for centrosome clustering. Overall, the experiments are straight-forward and rely almost exclusively on in vitro reconstitution with purified GFP-HSET and IFT52/70. For the most part the experiments are clearly documented, but there are a number of missing controls and questions about the specificity of the interactions. In addition, given that the effects of HSET oligomerization on HSET processivity has been shown previously as well as the interaction with IFT52/70, I question whether the advances really bring new biological insight into either the mechanisms of HSET motility or how HSET motility is utilized in centrosome clustering. The work is probably more suitable for a specialized journal because the experiments required to increase the impact are challenging, for example, identifying the key sites of interaction between HSET and IFT52/70 and then demonstrating that this interaction is indeed what is critical for centrosome clustering. Below are some comments that may be useful for the authors to improve the manuscript.
1. In the pulldowns in Figure 1, there is a high background of binding non-specifically to the beads or to the GFP, raising concerns about the specificity of the interaction. Panel d is missing the IFT46 channel control in the imaging.
2. In the motility experiments the authors should be using dot plots so that the reader can see the individual data and the spread of the data. They can be color-coded by experiment to show the day-to-day variability.
3. In Figure 2, the authors show complex formation, and the HSET/IFT52/70 complex is only 5% of the complex? How can this complex thus be attributed to the behaviors the authors report? This seems unlikely.
4. Figure 2F needs a more rigorous analysis of curve fits and statistical comparisons between the fit curves rather than just comparing the proportion of complexes from the bars in the histogram.
5. I think I may be confused by the experiment in Figure 3 with the GFP-HSET and the Kip2-RFP. Are both motors combined together with the IFT52/70? I am trying to understand how IFT52/70 gets to the minus ends of the MTs in the sample with the Kip2RFP. This would be a lot easier to interpret if the authors would just show each channel individually and then the merged image of all channels. I feel that in several experiments I am missing a channel to see all the controls/conditions.
6. In Figure 3e the authors plot the means plus SEM, but then use a mann-whitney test, which is a rank/sum test and not appropriate for comparing the means. Same is true in Figure 5c. It is also not clear how the individual slopes were obtained in this figure.
7. Maybe I am missing it, but I am struggling to find the control of each of the IFT proteins with MTs alone to see what their binding pattern looks like.
8. In Figure S1 legend, the bleaching steps should refer to a single GFP not a single GFP-HSET monomer; the HSET is an obligate dimer, but a single GFP in it could be bleached. Also why in the violin plot is the fluorescence equivalent to a tetramer for HSET alone (two dimer) and then the IFT52/70 looks like it only it increases to the level of three dimers?
9. Even after staring at the movies, I have no idea what the color coding in Figure S2b represents. Also, for the movies, they lack labels, time stamps and scale bars. It would also be much more helpful if the movies were presented in side-by-side panels for the conditions that are to be compared so the reader can judge the magnitude of the differences.
10. In Supplementary panel 3D, the authors report mean + SEM of n=2 experiments. This is statistically incorrect as one needs at least 3 points to do a proper SD, and SEM is SD/square root of n. While SEM makes for nice small error bars in a figure, it is often used inappropriately.
11. In the discussion, the authors state that this work provides a mechanistic explanation for how IFT proteins cluster centrosomes. This is a vast overstatement from the results presented.
12. In the discussion the authors propose that the mechanism involves oligomerization of HSET, and yet the biochemistry

shows very little complex formation.

Reviewer #2

(Remarks to the Author)
Using purified proteins and in vitro reconstitution, this study demonstrates that IFT52/70 directly binds to HSET, resulting in its oligomerisation and processive motility along microtubules. This interaction increases the motor's capacity to slide antiparallel microtubules and promotes the formation of active microtubule networks that evolve into asters.

This is a solid and valuable study that provides a mechanistic explanation for how IFT proteins contribute to centrosome clustering in dividing cells harbouring extra centrosomes. I support publication in Communications Biology, provided that the authors address the following relatively minor issues:

1. HSET clustering by IFT52/70 during processive motility is convincing, but it remains unclear whether IFT52/70 itself also oligomerises during movement. This should be tested, for example by quantifying the fluorescence intensity of labelled IFT52/70.
2. Figure 1: Please provide run-length data for HSET in the presence and absence of IFT52/70. This would most clearly indicate the extent to which the IFT proteins enhance motor processivity.
3. The rationale for using mouse IFT52 together with human IFT70 should be explained. The use of heterogeneous proteins needs justification.
4. Figure 2b–d (mass photometry): It is not entirely clear if the ~60 kDa peak corresponds to background signals in 2c and 2d. The peak profiles appear different from the "buffer alone" control in Supplementary Figure 1b. Please present the control data alongside the main panels, using consistent y-axis scaling.
5. Time stamps are missing from most of the movies and should be included.

Reviewer #3

(Remarks to the Author)
Guesdon et al. present a rigorous in vitro reconstitution study dissecting how the IFT52/70 subcomplex regulates the kinesin-14 motor HSET. Using biochemical interaction assays, mass photometry, single-molecule imaging, and microtubule network contraction assays, the authors show that IFT52/70 binds directly to HSET, promotes its oligomerization, increases its processive motility, and enhances its ability to slide and organize microtubules into polarized structures. These findings provide a mechanistic explanation for earlier cellular observations linking IFT proteins to HSET-dependent centrosome clustering.

Compared to the manuscript uploaded to bioRxiv and a version I previously reviewed, the present submission has been substantially strengthened, both experimentally and conceptually. The addition of mass photometry, cooperative activation analysis, expanded negative controls, and a clearer articulation of the findings within the kinesin-14 literature makes the study more rigorous and compelling. The revised manuscript now also provides strong mechanistic support for the model in which IFT52/70 promotes HSET oligomerization, resulting in enhanced processive motility.

In addition, the writing is clearer, the mechanistic framework is more coherent, and speculative statements have been appropriately removed. The manuscript now accurately reflects known aspects of kinesin-14 N-terminal tail interactions, including recent findings that these interactions act as specific mechanical brakes. The authors also appropriately connect their in vitro findings to known cellular phenotypes without overstating this relationship.

My only previous comment concerned the testing of a potential autoinhibition model. While the authors have not addressed this experimentally, they now explicitly step back from this hypothesis and no longer present it as a mechanistic conclusion, which is appropriate.

In conclusion, I recommend acceptance of the manuscript as submitted.

Version 1:

Reviewer comments:

Reviewer #1

(Remarks to the Author)
In this revised manuscript the authors report how the IFT52/70 complex impacts HSET motor activity through in vitro reconstitution. The authors have done an outstanding job responding to the concerns of all three reviewers, and the paper is much stronger than the previous submission. Personally, I still think that the evidence that the reported findings reflect the mechanism by which HSET helps in centrosome clustering in cells is slim, the work as presented by the authors is solid and hopefully their future studies will address these mechanistic questions. In addition, from reading the critiques, it appears that the original submission to Communications Biology was already a revised paper, which the authors have further revised. In the best interests of science and the work of the authors, I strongly recommend acceptance and publication of the current

paper.

Reviewer #2

(Remarks to the Author)
The authors have satisfactorily addressed all of my comments in the revision.

Open Access This Peer Review File is licensed under a Creative Commons Attribution 4.0 International License, which permits use, sharing, adaptation, distribution and reproduction in any medium or format, as long as you give appropriate credit to the original author(s) and the source, provide a link to the Creative Commons license, and indicate if changes were made.
In cases where reviewers are anonymous, credit should be given to 'Anonymous Referee' and the source.
The images or other third party material in this Peer Review File are included in the article’s Creative Commons license, unless indicated otherwise in a credit line to the material. If material is not included in the article’s Creative Commons license and your intended use is not permitted by statutory regulation or exceeds the permitted use, you will need to obtain permission directly from the copyright holder.
To view a copy of this license, visit https://creativecommons.org/licenses/by/4.0/

We would like to thank the reviewers for their useful comments and for the suggested experiments/analysis that helped reinforce our conclusions and strengthened the manuscript. The revised version of the manuscript now includes additional experiments, figures and text modifications that we believe would address their points.
Below is a point by point response to the reviewers.

Reviewer #1 (Remarks to the Author):

In the current manuscript the authors examine the mechanisms by which the IFT52/70 complex stimulates HSET activity by enhancing processivity and inducing oligomerization of HSET in vitro. They postulate that this mechanism is critical for centrosome clustering. Overall, the experiments are straight-forward and rely almost exclusively on in vitro reconstitution with purified GFP-HSET and IFT52/70. For the most part the experiments are clearly documented, but there are a number of missing controls and questions about the specificity of the interactions. In addition, given that the effects of HSET oligomerization on HSET processivity has been shown previously as well as the interaction with IFT52/70, I question whether the advances really bring new biological insight into either the mechanisms of HSET motility or how HSET motility is utilized in centrosome clustering. The work is probably more suitable for a specialized journal because the experiments required to increase the impact are challenging, for example, identifying the key sites of interaction between HSET and IFT52/70 and then demonstrating that this interaction is indeed what is critical for centrosome clustering. Below are some comments that may be useful for the authors to improve the manuscript.

1. In the pulldowns in Figure 1, there is a high background of binding non-specifically to the beads or to the GFP, raising concerns about the specificity of the interaction.
Pull-down conditions have been optimized (increased NaCl concentration, from 75 mM to 150 mM, increased reaction volume, from 40 µl to 100 µl). Results now show minimal non-specific binding. Figures and Methods have been modified accordingly.

Panel d is missing the IFT46 channel control in the imaging.
We have now updated all images on panel d to include the IFT46 control as requested.

2. In the motility experiments the authors should be using dot plots so that the reader can see the individual data and the spread of the data. They can be color-coded by experiment to show the day-to-day variability.
The data presented in Figure 1e are the number of events (a processive run of HSET alone or HSET associated to IFT46 or IFT52/70) per µm of microtubule per minute. The total number of events were divided by the total length of microtubule on which the events occurred divided by the imaging time when the events occur, so individual events cannot be plotted as individual values. Three independent experiments were done and they are represented as 3 individual black dots. The figure legend has been modified to present this information.

3. In Figure 2, the authors show complex formation, and the HSET/IFT52/70 complex is only 5% of the complex?
We agree that HSET-IFT52/70 complex represent a small proportion (5%) of all the proteins in solution. However, in the condition with HSET and IFT52/70 all the peaks containing IFT52/70 represent 13% of all proteins (8% for IFT52/70 alone and 5% for IFT52/70 associated to HSET), IFT52/70 associated with HSET thus represent 38% of all IFT52/70 present.
How can this complex thus be attributed to the behaviors the authors report? This seems unlikely.
Two facts may explain how the relatively small proportion of HSET-IFT52/70 complexes detected by mass photometry can lead to the behavior we studied using microscopy approaches.

First, binding to microtubule may strengthen HSET-IFT52/70 interaction stability since complex formation is observed in solution by mass photometry and all behaviors are assessed on microtubules.
Second, TIRF microscopy analysis may allow better detection of higher order complexes. The mass photometry assay detects complexes made of one HSET dimer and two IFT52/70, and also suggest some higher order complexes around 760 kDa (Supplementary Figure 1c) but we decided not to qualify them as proper peak/complex due to limited signal intensity (low number of counts). In contrast, TIRF microscopy allows the detection of higher order complexes that constitute the majority of the HSET-IFT52/70 complexes counted on microtubules (Figure 2g). Those higher order HSET-IFT52/70 complexes are more processive and accumulate more at microtubule ends (Figure 3). We propose that they are responsible for the increased microtubule organization activity of HSET (compared to HSET alone) described in Figure 4 and 5.
Differences in detection between mass photometry and TIRF approaches are commented in the discussion (page 10).

4. Figure 2F needs a more rigorous analysis of curve fits and statistical comparisons between the fit curves rather than just comparing the proportion of complexes from the bars in the histogram.
As requested, we re-analyzed the data presented in former Figure 2f (now Figure 2g) and fit curves obtained using non-linear regression with a gaussian fit were added. Multiple unpaired t-test analysis on the best fit values for the mean number of GFP-HSET dimer per particle indicates a significant difference ($p<0,05$) for the distribution of the two datasets. Similarly, a Chi ^{2} test on the distribution of the data between HSET and HSET-IFT52/70 conditions also indicates a significant difference ($p<0,0001$). Of note, we have now excluded the run length of less than 1 μm from the new analysis (to match the data set analyzed in Fig. 1e,f), statistical tests with or without removing those short run length give similar results. The result section figures and figure legend have been modified accordingly.

5. I think I may be confused by the experiment in Figure 3 with the GFP-HSET and the Kip2-RFP. Are both motors combined together with the IFT52/70? I am trying to understand how IFT52/70 gets to the minus ends of the MTs in the sample with the Kip2RFP.
Kip2-RFP and the HSET-IFT52/70 complex are flown sequentially in the same chamber:
- First, Kip2 is flown in the chamber and accumulates at the plus end. The chamber is then washed with BRB80 to remove unbound Kip2.
-Second, HSET incubated with IFT52/70 are flown in the chamber and accumulates at the end opposite to where Kip2 localized, therefore indicating a minus-end accumulation of HSET-IFT52/70 complex.
This is now detailed in the method section.

This would be a lot easier to interpret if the authors would just show each channel individually and then the merged image of all channels. I feel that in several experiments I am missing a channel to see all the controls/conditions.
As requested, Figure 3 panel b has been reorganized to show IFT52/70, Kip2-RFP and GFP HSET individually as well as the merged of HSET and Kip2 and the merged of IFT52/70 and Kip2. Following the reviewer request (point 1, 5 and 7) control channels have been added either in the main figure or the Supplementary Figures.

6. In Figure 3e the authors plot the means plus SEM, but then use a mann-whitney test, which is a rank/sum test and not appropriate for comparing the means.
This is now modified and an unpaired Student's t-test was done to compare the means. The significances of the test are unchanged. The legend was modified accordingly.
Same is true in Figure 5c. It is also not clear how the individual slopes were obtained in this figure.

The slopes presented in Figure 5c are calculated using a simple linear regression of the values of image contrast between 8 and 11 minutes, this is now described in the figure legend. The measured values during this time interval are presented on the graph. This is now better explained in the figure legend. An unpaired Student's t-test was done and the graph was updated accordingly.

7. Maybe I am missing it, but I am struggling to find the control of each of the IFT proteins with MTs alone to see what their binding pattern looks like.
IFT52/70 and IFT46 alone do not bind to microtubules. We now provide representative images of IFT46 and IFT52/70 in presence of microtubules in Supplementary Figure 1b.

8. In Figure S1 legend, the bleaching steps should refer to a single GFP not a single GFP-HSET monomer; the HSET is an obligate dimer, but a single GFP in it could be bleached.
This has been corrected.
Also why in the violin plot is the fluorescence equivalent to a tetramer for HSET alone (two dimer) and then the IFT52/70 looks like it only it increases to the level of three dimers?
We used this violin plot to show the distribution HSET and HSET+IFT52/70 particles. In the HSET alone control the majority of particles contain either one (750 a.u.) or two HSET dimer while in the condition with IFT52/70 the majority of particles contain two, three or more than three dimers. Detailed numbers of each category are represented in Figure 2g and the new fit curves and statistical analysis requested in point 4 now reflect the statistical differences between control and IFT52/70 conditions.

9. Even after staring at the movies, I have no idea what the color coding in Figure S2b represents. Also, for the movies, they lack labels, time stamps and scale bars. It would also be much more helpful if the movies were presented in side-by-side panels for the conditions that are to be compared so the reader can judge the magnitude of the differences.
We agree with reviewer 1 that color-coded images were difficult to interpret. Therefore, figure S2b has been changed to present still images at successive times points. The movies corresponding to Supplementary Figure 2 (movies 4 and 5) have been labelled and mounted side by side as requested.

10. In Supplementary panel 3D, the authors report mean + SEM of n=2 experiments. This is statistically incorrect as one needs at least 3 points to do a proper SD, and SEM is SD/square root of n.
We have done the experiment in triplicate. Supplementary Figure 3 panels c and d were updated

While SEM makes for nice small error bars in a figure, it is often used inappropriately.
We use S.E.M to visualize how precise are the mean of our sample compared to the true population mean. When possible, we are showing in the figure the individual data distribution (using dot plots, or combined dot plots and histograms) to show data variability. We updated Figure 2a where data spread was not shown by adding individual measurements to the histogram.

11. In the discussion, the authors state that this work provides a mechanistic explanation for how IFT proteins cluster centrosomes. This is a vast overstatement from the results presented.
We have edited the text in the discussion (page 10, 12) to take in consideration the reviewer's comment.

12. In the discussion the authors propose that the mechanism involves oligomerization of HSET, and yet the biochemistry shows very little complex formation.
As discussed in point 3, we acknowledge that HSET-IFT52/70 represent a small proportion of all proteins when studied in solution using mass photometry. Significant increase in

oligomerization of HSET in presence of IFT52/70 is observed using TIRF microscopy (Figure 2g). We mentioned those differences in the discussion including potential explanations (pages 10 and 11).

Reviewer #2 (Remarks to the Author):

Using purified proteins and in vitro reconstitution, this study demonstrates that IFT52/70 directly binds to HSET, resulting in its oligomerisation and processive motility along microtubules. This interaction increases the motor's capacity to slide antiparallel microtubules and promotes the formation of active microtubule networks that evolve into asters.

This is a solid and valuable study that provides a mechanistic explanation for how IFT proteins contribute to centrosome clustering in dividing cells harbouring extra centrosomes. I support publication in Communications Biology, provided that the authors address the following relatively minor issues:

1. HSET clustering by IFT52/70 during processive motility is convincing, but it remains unclear whether IFT52/70 itself also oligomerises during movement. This should be tested, for example by quantifying the fluorescence intensity of labelled IFT52/70.
To address the reviewer comment, we correlated the fluorescent intensity of the GFP-HSET to the fluorescence intensity of IFT52/70-Alexa Fluor 647 (Rebuttal Figure 1). This analysis indicates a good correlation between IFT52/70 Alexa Fluor 647 and GFP-HSET signals (R=0,813). Thus, it indicates that the number of IFT52/70 increases proportionally to the number of GFP-HSET within the processive HSET-IFT52/70 particles. This suggest that IFT52/70 does not oligomerized within the particles independently of GFP-HSET. Unfortunately, we were not able to determine the exact number of IFT52/70 per particle due to the high photostability of Alexa Fluor 647 that prevents its photobleaching in our imaging condition.

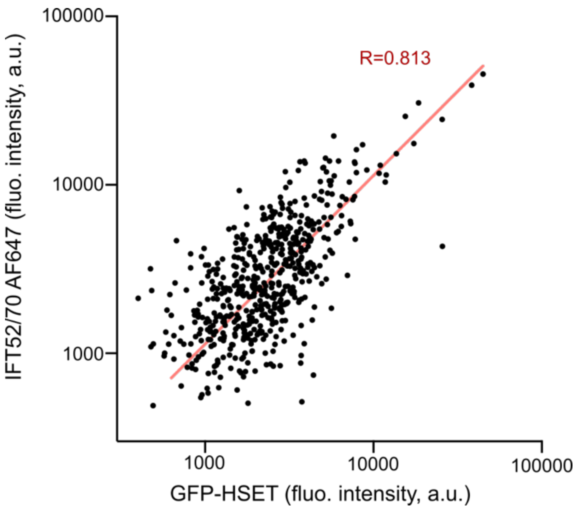

Rebuttal Figure 1. IFT52/70 fluorescence intensity increases proportionally to HSET fluorescence intensity. The red line indicates a simple linear regression fit to the data. R indicates the Pearson correlation coefficient.

2. Figure 1: Please provide run-length data for HSET in the presence and absence of IFT52/70. This would most clearly indicate the extent to which the IFT proteins enhance motor processivity.
We added a panel to Figure 1 (panel f) to show HSET particles processivity and edited the text accordingly.

3. The rationale for using mouse IFT52 together with human IFT70 should be explained. The use of heterogeneous proteins needs justification.
We previously showed that mIFT52 and hIFT70 in cells can form functional heterogeneous protein complexes since mIFT52 can rescue hIFT52 depletion in human RPE-1 cells (Figure1E of PMID 32270908). In addition, given the high sequence identity between mIFT52 and hIFT70 amino acid sequence (91.7% identity using Expasy SIM analysis) and the fact that they can form a dimer (Figure 2c) we decided to do our experiment using this heterogeneous protein dimer (hIFT70 from the genomic collection hORFeome database V8.1 and mIFT52 from the Lorentzen lab, Taschner et al, JCB, 2014; PMID25349261; hIFT52 was not available from the hORFeome database v8.1). Additional information regarding this point were added to the method section.

4. Figure 2b–d (mass photometry): It is not entirely clear if the ~60 kDa peak corresponds to background signals in 2c and 2d. The peak profiles appear different from the "buffer alone" control in Supplementary Figure 1b. Please present the control data alongside the main panels, using consistent y-axis scaling.
According to the reviewer comment, we changed the y-axis to display consistent scaling between figures. We also included the buffer only control to the main figure (Figure 2b).
The 60 kDa peak does not correspond to buffer background only. This peak could also contain monomeric IFT52 (67 kDa) or IFT70 (95 kDa) and contaminants such as peptides resulting from IFT52 or IFT70 degradation as well as HRV-3C which is present in IFT purifications (* in Figure 1a). In the case of GFP-HSET the peak could contain degradation products of GFP-HSET. This is now included in the text (page 5).

5. Time stamps are missing from most of the movies and should be included.
Times stamps were added to all movies. Of note, movies have been combined to show different conditions on the same movie or different channels on the same movie according to reviewer 1 request.

Reviewer #3 (Remarks to the Author):

Guesdon et al. present a rigorous in vitro reconstitution study dissecting how the IFT52/70 subcomplex regulates the kinesin-14 motor HSET. Using biochemical interaction assays, mass photometry, single-molecule imaging, and microtubule network contraction assays, the authors show that IFT52/70 binds directly to HSET, promotes its oligomerization, increases its processive motility, and enhances its ability to slide and organize microtubules into polarized structures. These findings provide a mechanistic explanation for earlier cellular observations linking IFT proteins to HSET-dependent centrosome clustering.

Compared to the manuscript uploaded to bioRxiv and a version I previously reviewed, the present submission has been substantially strengthened, both experimentally and conceptually. The addition of mass photometry, cooperative activation analysis, expanded negative controls, and a clearer articulation of the findings within the kinesin-14 literature makes the study more rigorous and compelling. The revised manuscript now also provides strong mechanistic support for the model in which IFT52/70 promotes HSET oligomerization, resulting in enhanced processive motility.

In addition, the writing is clearer, the mechanistic framework is more coherent, and speculative statements have been appropriately removed. The manuscript now accurately reflects known aspects of kinesin-14 N-terminal tail interactions, including recent findings that these interactions act as specific mechanical brakes. The authors also appropriately connect their in vitro findings to known cellular phenotypes without overstating this relationship.

My only previous comment concerned the testing of a potential autoinhibition model. While the

authors have not addressed this experimentally, they now explicitly step back from this hypothesis and no longer present it as a mechanistic conclusion, which is appropriate.

In conclusion, I recommend acceptance of the manuscript as submitted.
